# Bioactivity of Chitosan-Based Particles Loaded with Plant-Derived Extracts for Biomedical Applications: Emphasis on Antimicrobial Fiber-Based Systems

**DOI:** 10.3390/md19070359

**Published:** 2021-06-23

**Authors:** Joana C. Antunes, Joana M. Domingues, Catarina S. Miranda, A. Francisca G. Silva, Natália C. Homem, M. Teresa P. Amorim, Helena P. Felgueiras

**Affiliations:** Centre for Textile Science and Technology (2C2T), University of Minho, Campus de Azurém, 4800-058 Guimarães, Portugal; joana.domingues@2c2t.uminho.pt (J.M.D.); catarina.miranda@2c2t.uminho.pt (C.S.M.); pg40181@alunos.uminho.pt (A.F.G.S.); natalia.homem@2c2t.uminho.pt (N.C.H.); mtamorim@det.uminho.pt (M.T.P.A.); helena.felgueiras@2c2t.uminho.pt (H.P.F.)

**Keywords:** chitosan, plant extracts, drug delivery systems, nanoparticles, bioactive, electrospun fibers, medical textiles

## Abstract

Marine-derived chitosan (CS) is a cationic polysaccharide widely studied for its bioactivity, which is mostly attached to its primary amine groups. CS is able to neutralize reactive oxygen species (ROS) from the microenvironments in which it is integrated, consequently reducing cell-induced oxidative stress. It also acts as a bacterial peripheral layer hindering nutrient intake and interacting with negatively charged outer cellular components, which lead to an increase in the cell permeability or to its lysis. Its biocompatibility, biodegradability, ease of processability (particularly in mild conditions), and chemical versatility has fueled CS study as a valuable matrix component of bioactive small-scaled organic drug-delivery systems, with current research also showcasing CS’s potential within tridimensional sponges, hydrogels and sutures, blended films, nanofiber sheets and fabric coatings. On the other hand, renewable plant-derived extracts are here emphasized, given their potential as eco-friendly radical scavengers, microbicidal agents, or alternatives to antibiotics, considering that most of the latter have induced bacterial resistance because of excessive and/or inappropriate use. Loading them into small-scaled particles potentiates a strong and sustained bioactivity, and a controlled release, using lower doses of bioactive compounds. A pH-triggered release, dependent on CS’s protonation/deprotonation of its amine groups, has been the most explored stimulus for that control. However, the use of CS derivatives, crosslinking agents, and/or additional stabilization processes is enabling slower release rates, following extract diffusion from the particle matrix, which can find major applicability in fiber-based systems within ROS-enriched microenvironments and/or spiked with microbes. Research on this is still in its infancy. Yet, the few published studies have already revealed that the composition, along with an adequate drug release rate, has an important role in controlling an existing infection, forming new tissue, and successfully closing a wound. A bioactive finishing of textiles has also been promoting high particle infiltration, superior washing durability, and biological response.

## 1. Chitosan

**Figure 1:**
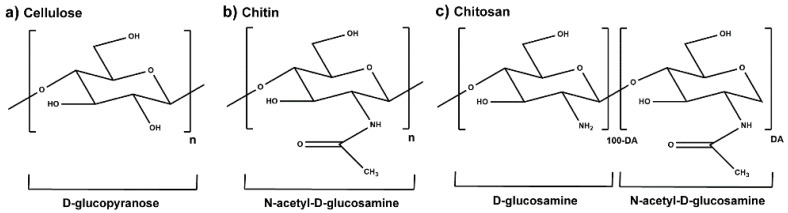
Chemical structure of the molecular units of (**a**) cellulose, (**b**) chitin, in the absence of partial deacetylation, and (**c**) partially acetylated CS characterized by the DA (adapted from [3,5,6]).

Chitin is the second most abundant biologically derived polymer worldwide, after cellulose [1]. It is the primary structural component of the exoskeleton of shrimps, crabs, lobsters, and squid pens, and is present in smaller amounts in the cell walls of some fungi and yeast and in plants [2]. This polysaccharide has a chemical structure similar to that of cellulose, with hydroxyl groups at position C-2 replaced by acetamido groups [3]. Chitosan (CS) is mainly obtained by partial deacetylation of chitin, under high temperatures and alkaline conditions [1,4], when the degree of acetylation (DA, molar fraction of N-acetylated units) is lower than ≈50%. Glucosamine and N-acetylglucosamine are connected through a 1,4-glycosidic bond to form the skeleton of CS, which leads to a linear polymeric structure (Figure 1) [3,5].

CS’s molecular weight (Mw) and the DA are its main structural parameters influencing the overall behavior of the polymer as a biomaterial [2,7]. CS with a wide range of DA and Mw can be found commercially (DA < 35% and M_w_ between 10 and 800 kDa), being widely accepted that low Mw is below 50 kDa, medium Mw is between 50 and 150 kDa, and high Mw is superior to 150 kDa [8]. CS is soluble in nearly all diluted aqueous acidic solutions and insoluble in water, concentrated acid, alkali, alcohol, and acetone and in common organic solvents. The polymer can be degraded enzymatically, through chemoenzymatic means, recombinant approaches, and physical means such as electromagnetic radiation and sonication. In humans, in vivo degradation of CS is thought to be primarily due to the activity of lysozymes (present in articular cartilage, liver, plasma, saliva, tears, and milk) and bacterial chitosanolytic enzymes (e.g., chitosanase) that have been identified in human tissues of the gastrointestinal tract and lung. These enzymes hydrolyze both glucosamine and acetylated residues, leading to polymer erosion into a suitable size for renal clearance [2,9].

CS is regarded as a nontoxic and a biologically compatible polymer, extensively studied for multiple biomedical applications including the formulation of small-scale drug delivery systems [2,10]. Among its numerous attractive features, mostly connected to its peripheral groups, notably its primary amines and hydroxyl groups, the polymer inherently exerts mucoadhesive, haemostatic, chemoattractive, regenerative, analgesic, antioxidant, and immunomodulatory traits [2,11,12,13]. Its mucoadhesiveness results in transient opening of the tight junctions between epithelial cells of the intestinal mucosal barrier to enhance permeation of drugs, proteins, and food nutrition [14]. Its resemblance to human proteoglycans, thus being prone to molecular recognition by living cells or tissues, makes it an appealing regeneration enhancer [2]. The polysaccharide’s terminal moieties react with the unstable free and reactive oxygen species (ROS) stabilizing them, namely, CS with low DA and Mw [7]. A low DA also encourages an anti-inflammatory response [15,16,17], with a high DA favouring a pro-inflammatory phenotype that can be useful to counteract cancer cell invasion [18,19,20]. Moreover, CS is endowed with antimicrobial capacity and enhanced ability to regulate gut microbiota towards homeostasis [2,11,12,13,21]. CS oligosaccharide (DA = 12% and Mw < 1 kDa) supplementation (while dispersed in water) has been shown to decrease blood glucose levels and reverse the insulin resistance of diabetic mice, together with having higher intestinal integrity, and suppressed inflammation and lipogenesis, thereby contributing to gut microbial balance [22]. CS nanoparticle (NP; diameter (d) ≈ 50 nm, built with CS with DA = 5% and Mw = 220 kDa through undisclosed methodology) intake exerted a positive influence over the composition of colonic microbiota of weaned pigs [23]. Gut dysbiosis enables pathogens to dominate the gut, mostly bacteria [24]. Hence, it is worth mentioning that CS’s antibacterial activity is particularly interesting. It is mainly of electrostatic nature, when its amine groups are protonated (which traditionally occurs at 9.5 < pH < 6.5, depending on its DA [25]) [26,27]. Two mechanisms of antibacterial action have been proposed: presence on the cell surface, forming a polymer layer preventing substance exchange, interfering with nutrient intake; or CS of lower Mw reaching the intracellular environment, adsorbing electronegative substances thereby disrupting the physiological activity of bacteria and killing them. Literature also highlights a concentration-dependent antibacterial effect of CS [4,6,26]. However, the effect of the polysaccharide can be limited in basic, or even neutral, environments [25,26,27]. Consequently, a large number of CS derivatives are being developed, given that its amino and hydroxyl groups confer the polymer with a high chemical versatility that has been widely explored to maximize the polymer processability, solubility, pH-responsiveness over a larger pH range, as well as its antimicrobial efficiency [10]. CS derivatives are easily obtained [3,28], including amine (N-modified) and hydroxyl (O-modified) group substitution by acylation, carboxylation, alkylation, and quaternization, among others [10,29]. Table 1 reveals the latest trends (between 2020 and 2021) regarding CS and CS derivatives used as antibacterial agents for biomedical applications. Regulatory approval for the use of CS and its derivatives in the highlighted fields has required material characterization and production consistency, functionality, specifications of the product, material and product safety profile and analysis using validated methods [30]. CS continues to be widely explored for its antibacterial features, being incorporated into increasingly complex architectures to attempt solving multivalent clinical needs. However, despite knowing that a particular range of DA and/or Mw may enhance CS’s antibacterial capacity [27], the choice behind CS’s batch selection remains poorly justified, with the polymer’s inherent properties being poorly characterized as well. However, efforts clearly benefit from CS’s chemical versatility to create polymeric derivatives with ingenious capabilities, providing added value towards multiple biomedical applications.

## 2. Plant-Derived Biomolecules

Plant extracts are widely used as natural drugs in conventional medicine, given their high availability from nature (e.g., seeds, bark, wood, roots, leaves, flowers, and fruits), bioactivity, operating facility, reduced capital costs, and scalability [65,66]. Plants synthetize a large panoply of structurally different compounds such as simple phenols and phenolic acids, quinones, flavonoids, tannins, coumarins, terpenes and terpenoids, alkaloids, lectins and polypeptides, among other phytochemicals, each having a specific and distinct role in the plant’s bioactivity [66,67,68,69,70,71]. Some, such as terpenoids, also give plants their odors; others (quinones and tannins) offer to plants their pigmentation [69]. These biomolecules can exert strong antioxidant, anticancer, anti-inflammatory, and antimicrobial properties at their site of action [14,72,73,74,75]. Their ability to inactivate free radicals is mostly mediated by phenolic biomolecules within its composition, namely the hydrogen atoms of the adjacent hydroxyl groups (o-diphenol), the double bonds of the benzene ring, and the double bond of the oxo functional group of some flavonoids. They reduce tissue lipid oxidation, this way delaying aging, decreasing inflammation, oxidative stress, as well as the chances of developing some diseases, namely cardiovascular pathologies (e.g., myocardial infarction and atherosclerosis), cancer, metabolic (e.g., diabetes) and neurological disorders (e.g., depression) [14,70,76]. Plant-based metabolites act as defense mechanisms against invasive microorganisms, insects, and herbivores. They wield antibacterial activity via multiple mechanisms, acting in consonance for increased host protection. Their chemical versatility has additionally enabled the synthesis of a large variety of functionalized skeletons. Modes of action are variable, yet potent [77,78,79,80]. Inhibition of cell wall synthesis, permeabilization and disintegration of bacterial peripheral layers, restriction of bacterial physiology, oxygen uptake and oxidative phosphorylation, efflux pump inhibition, modulation of antibiotic susceptibility, biofilm inhibition, hindrance of the microbial protein adhesion to the host’s polysaccharide receptors, and attenuation of bacterial virulence, are known and acclaimed mechanisms of action of such elements [67,69,70,81]. Compounds such as lectins even allow specific recognition and reversible interaction to either free carbohydrates or glycoconjugates, without modifying their structure. They may form ion channels in the microbial membrane or inhibit adhesion of microbial proteins to host polysaccharide receptors. Hence, they are capable of precipitating polysaccharides and glycoproteins or agglutinating cells [82,83,84,85]. Overall, these changes are mostly induced by hydrophobic effects, covalent binding and hydrogen binding of their phenolic compounds [69]. The multitarget action of plant extracts, unlikely to induce resistance [86], has the potential to surpass the current clinical failures posed by traditional antibiotics [66]. Table 2 illustrates the main classes of antibacterial plant constituents based on the categorization published by Cowan [69], including the representation of chemical structures of relevant examples. For instance, gallic acid (a phenolic acid), while loaded into CS-based NPs and dispersed within collagen and fibrin hydrogels [87], has shown an excellent DPPH (2,2-Diphenyl-2-picryl hydrazyl hydrate) radical scavenging activity even at the lowermost concentration of 0.05 mg/mL, strongly contributing for a faster re-epithelialization and wound contraction, qualities that are highly valued for wound dressing applications. In another study [88], thyme-essential-oil-loaded CS NPs and nanocapsules, rich in thymol and carvacrol (simple phenols), exhibited an antibacterial action dependent on thymol and carvacrol release rate, with 100% phenol release in 5 h (rather than 10 h) evoking 50% larger ZoIs, thus reinforcing their importance in the field. Authors indicated that studies related to mechanism of action on bacteria were ongoing. A final example described cinnamaldehyde combination with CS in the form of NPs via Schiff reaction between the free amine groups of CS and the aldehyde group of the phenylpropanoid [89]. It substantially enhanced CS’s antibacterial capacity, additionally improving the stability of the CS NPs. The bacterial growth inhibition was 33–34% higher for grafted CS than for the unmodified polysaccharide-based NPs. Lectins and polypeptides were excluded from the table, given that they have more complex structures than the other cited classes. Regardless, these proteins or glycoproteins are often positively charged, with disulphide bonds. Concanavalin A and galectin-1 are well-known examples, having as ligands Manα1-OCH_3_ and Gal(β1→4)Glc, respectively [82,83,84,85,90].

## 3. Chitosan-Based Small-Scaled Particles Loaded with Plant-Derived Biomolecules

Most of the chemical components of plant extracts are, in general, volatile and susceptible to temperature, light incidence, oxygen- and/or moisture-induced degradation, thereby losing efficacy [121,122]. In some cases, these can even induce toxicity and allergic reactions [123]. Small-scaled particles, as drug reservoirs, can bypass the later issues due to their capacity to control drug delivery and provide effective solutions [121,122,123].

CS has already been the object of a vast number of very interesting studies, as NP, MP, particle-, film-, or coating-layer component [34,124,125,126,127,128,129] or even as reducing agent of inorganic NPs [130]. However, these formulations have excluded plant extracts from their composition. Much has also been published on the use of plant extracts as reducing agents for inorganic NP synthesis, namely silver, gold, zinc, or copper oxide NPs [131,132,133,134]. However, organic NPs, templated upon natural or synthetic organic molecules, are more easily recognized by the host and biodegraded. CS has been extensively explored as a carrier component of organic drug delivery systems (mostly nanoparticles, NPs) for load, and release, of plant-derived compounds [135], with hydrophobic biomolecules being traditionally encased by a CS-based shell, and hydrophilic biomolecules entrapped within the CS-containing matrix.

**Figure 2:**
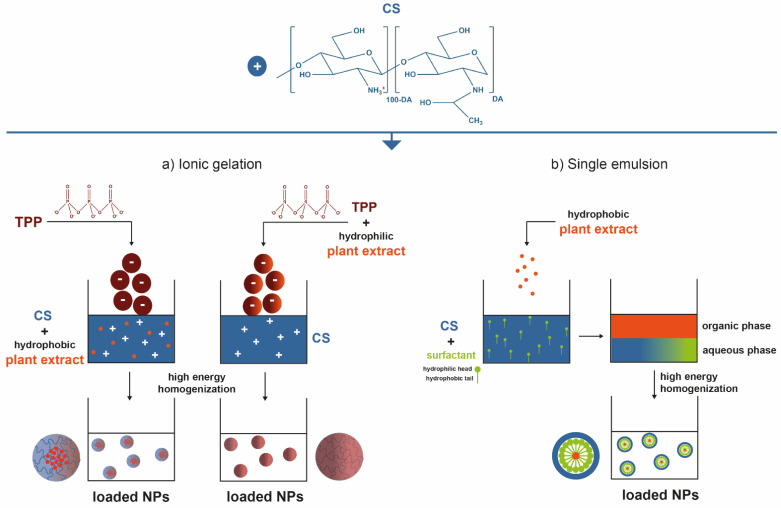
Key steps involved in the preparation of plant-extract-loaded CS-based NPs by (**a**) ionic gelation and (**b**) simple emulsion techniques [2,66,144,145,152].

Nanoparticulate systems are colloidal-sized particles with diameters ranging from 1 to 1000 nm [136,137,138]. Their size offers a high surface/volume ratio and the correlation with structural sizes of biological components: they are small enough to pass through biological barriers, internalize target cells, and influence a number of cellular processes [139,140,141]. Loaded NPs can protect the cargo from biodegradation, thus retaining their bioactivity, extend circulation times, enable their controlled release, and ensure their efficacy at the target site, using lower doses than if they were to be used in free form [123,142]. Depending on the method employed for their preparation, nanospheres—matrix-like systems in which the drug is dispersed within the polymer chains—or nanocapsules—vesicular systems that are formed by a drug-containing liquid core (aqueous or lipophilic) surrounded by a single polymeric membrane, can be obtained [143,144,145]. Ionic gelation is the most commonly described procedure for CS-based NP production. In short, CS has the ability to function as a polyelectrolyte, as it is a polymeric macromolecule with charged or chargeable groups (particularly its primary amine groups) when dissolved in polar solvents (predominantly water) [2]. Ergo, ionic gelation is a self-assembly process driven by electrostatic interactions between aqueous solutions of charged macromolecules such as CS and small molecules (like tripolyphosphate, TPP) carrying opposite electrical charges [66,146,147]. It is an easy, versatile, low-cost technology, requiring a simple and easily scaled-up apparatus, enabling multiple biomolecules incorporation with high efficiency, stability, and controlled release [2,148]. CS-based small-scale particles have also been broadly generated by emulsification methods. A single emulsion/solvent extraction method is another frequent example [143,144,145,146,147]. An emulsification protocol (exposure to high energy source: ultrasound, homogenizer, milling) implies mixing one liquid phase into another totally or partially immiscible by resorting to stabilizers like surfactants, which are able to reduce the interfacial tension between the two liquid phases to achieve stability [143,145]. Typically, a non-water-miscible organic solution of a hydrophobic drug is mixed with preformed polymers into an aqueous phase containing surfactants. Nano-sized organic solvent droplets are obtained, being templates for nanocarrier assembly. The non-aqueous phase is removed by evaporation under low pressure or vacuum or by solvent extraction using a large volume of water, leading to the formation of NPs dispersed in the water phase. Hence, formed NPs are then collected by centrifugation or filtration and washed with pure water or buffer solution to remove residual stabilizers and free drug, and freeze-dried for storage [144,145]. Alternatively, hybrid techniques like emulsification followed by ionic gelation can be pursued, so that the hydrophilic particle surface is further stabilized [73,75,149,150,151]. To encapsulate hydrophilic drugs, a double emulsion (water-in-oil-in-water) may be formed with the drug dissolved in the internal aqueous phase [145]. That, however, has not appear in published work. Figure 2 represents the most commonly employed processing methodologies to create small-scaled organic particles with CS as skeletal component and carrying plant extracts for enhanced biological effect.

**Figure 3:**
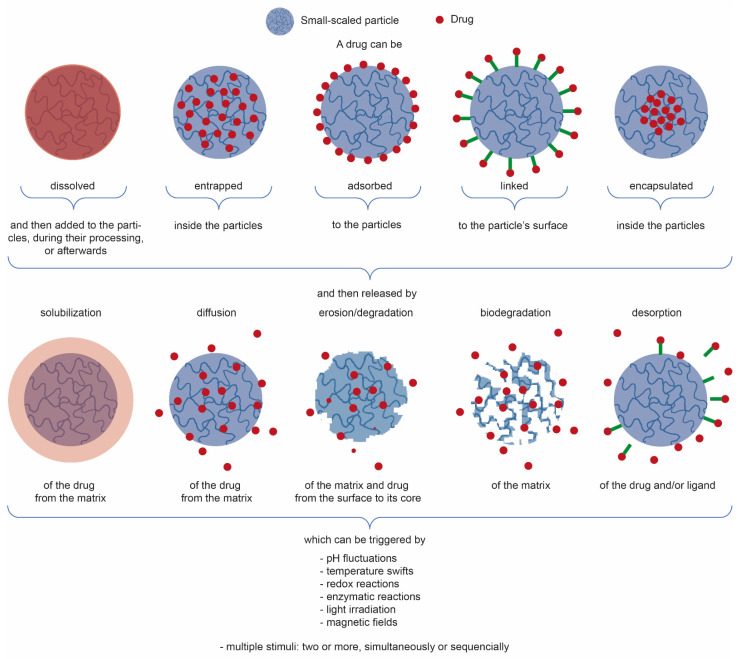
Simplified illustration of the main strategies used for drug conjugation with small-scale particles and drug release mechanisms, with the indication of the classic triggers responsible for their release from the particles [154,155,156].

Table 3 showcases relevant examples of plant-extract-loaded CS-based small-scale particles for the aforementioned biomedical applications. Most of the research is being done with low-medium Mw CS and 15 < DA < 25%, used as-received, and processed in the form of NPs, namely, using ionic gelation, emulsification, or the hybrid top-down and bottom-up approaches such as emulsification followed by ionic gelation. Integrated plant extracts are mostly hydrophobic in nature, and encapsulated (or entrapped, literature is unclear) within the NP matrix, even though some of it gets adsorbed to the NPs, and some affinity with CS through hydrogen bonding may also take place [34]. After a certain amount of time and under certain conditions, the latter traditionally suffers a burst release while the remainder of the extract gets released over a longer period of time. Following the electrostatically self-assembly methods of ionic gelation or polyelectrolyte complexation, pH change (as it occurs after NP incubation in physiological conditions) is the most appointed trigger for drug release, given that a higher pH will deprotonate the primary amines of the CS and feed NP matrix disintegration. Notwithstanding, if the NPs are further stabilized, for instance through the use of trimethylated CS derivatives that offer pH-independent cationic charges (increasingly evident with higher DS) [119,151,153] or emulsification [73,75,149,150,151], thereby reinforcing NP stability, the appointed release mechanisms are instead driven by diffusion, with a contained matrix swelling allowing the drug to traverse the NPs and leave them, which is preceded by drug desorption from the NP peripheral chains. Figure 3 illustrates and summarizes the main paths taken by a drug to be loaded onto or into small-scale particles (depending on the goal, mechanism, and kinetic of actuation, and of NP type), which can then be released from them in different manners and triggered or controlled by multiple stimuli, either acting alone or combined to function in parallel or one after the other.

One of the examples presented in Table 3 highlights CS-quinoline NPs loaded with quercetin, a hydrophobic anticancer plant extract against HeLa cells. The release of the anticancer drug is controlled by pH fluctuations and showed high cytotoxicity for cancer cell proliferation. The results also demonstrated the potential of these CS-based NPs crosslinked with quinoline derivatives for drug delivery of other therapeutic agents [74]. For application as dietary supplements, CS and poly (γ-glutamic acid) (γ-PGA, an edible polyamino acid) NPs were loaded with tea catechins, which are potent antioxidant polyphenolic compounds present in green tea. Following oral administration, the severe gastrointestinal tract environment poses severe hurdles to the bioactivity of these oxidation-sensitive compounds. Their encapsulation in NPs solves this problem, and the results showed an efficient pH-responsive release of tea catechins from the NPs in simulated gastrointestinal tract media, with an effective antioxidant activity [14]. In the context of medical textiles, an interesting example depicted emulsion-derived CS NPs crosslinked with cinnamaldehyde, an extract from cinnamon trees that is also a bactericidal agent. The results demonstrated antibacterial activity against *S. aureus* (Gram-positive) and *E. coli* (Gram-negative) bacteria. These NPs can coat medical textiles such as wound dressings or even other antimicrobial sustainable textiles (e.g., sports wears, home textiles, automotive sector) [89]. Another example presented CS emulsion-derived MPs encapsulating lemongrass or geranium essential oils (EOs) to act against biofilm formation led by *Candida albicans*, a commensal fungus yet a dangerous opportunistic pathogen in certain medical conditions. The minimum inhibitory concentration (MIC) values for loaded MPs were lower than for unloaded MPs and free EOs. The higher EO-loaded MP biofilm inhibition percentage demonstrated the efficiency of MPs against *C. albicans* biofilm formation and endurance. EO was released by a slow, and sustained, pH-sensitive diffusion process [72]. A final example within the table described CS/TPP NPs synthesized by ionic gelation with incorporated gallic acid, which is a plant polyphenolic compound with wound healing properties along with anti-inflammatory, antioxidant, anti-cancer, anti-diabetic, and neuroprotective activities. With the synthesized NPs, the authors advanced the preparation of collagen/fibrin scaffold infused with the gallic-acid-loaded CS NPs. The results showed increased collagen deposition, angiogenesis, epithelialization and fibroblast migration which culminated in accelerated wound contraction [87]. These results also demonstrated the potential of the CS NPs to be incorporated in other biomaterial-processed architectures with suitable properties to facilitate their practical application.

## 4. Biomedical Applications: Fiber-Based Systems

Numerous processing methodologies exist for polymer phase change from solution into solid-state fibres, forming continuous monofilament or multifilament yarns or, alternatively, short-length staple fibers subsequently blended with natural fibers (e.g., cotton or wool), or used by themselves to create scaffolding systems [169]. 3D printing and fiber spinning technologies (e.g., fiber extrusion spinning, melt-spinning, dry-spinning, wet-spinning, electrospinning) are considered the most prominent techniques in the biomedical field to generate such fibrous structures [169,170].

Electrospun nanofiber-based systems are particularly appealing [5]. Mats produced by electrospinning resemble the morphological structure of the extracellular matrix due to their nanoscale features, are endowed with large surface area per unit volume, and arranged in a highly interconnected porous architecture, able to easily incorporate biomolecules or NPs of interest [5,169,171]. Electrospinning is a simple, effective, and versatile method to yield fibrous structures with fiber diameters ranging between few nanometers to lower than one micrometer, a size that is difficult to attain using conventional spinning techniques. Compared to other techniques used for nanofiber production, such as phase separation, self-assembly, template synthesis, mechanical drawing, melt blowing, hydrothermal processing, centrifugal force spinning, and bicomponent extrusion, this method is the most effective in producing nanofibers with a homogeneous structure [5,172,173], thus being the method of choice for this particular purpose [169,171]. A polymeric solution is injected through a needle and directed at a collector (frequently a conductive aluminum plate, which generates nonwoven structures). Due to the high applied electrical field, the potential difference created between the needle and collector attracts the polymer to the later while allowing solvent evaporation to occur along the taken path. The polymeric solution is this way converted into nanofibers [171]. The use of different polymers, polymer blends, or nanocomposites made of organic or inorganic materials can modulate the chemical composition of electrospun membranes. Physical parameters and structures, such as fiber diameter, mesh size, porosity, texture, and pattern formation can also be maneuvered, thereby offering numerous possibilities towards electrospun scaffold design that can meet the demands of an intended application [171,174].

However, most of these fiber-based systems rely on fabrication techniques that heavily depend on manual intervention, hindering reproductivity and scaling-up, and leading to high manufacturing costs. Textile technologies are a viable alternative to those approaches, enabling the production of finely tuned, fiber-based complex constructs with high control over the design (e.g., size, shape, porosity and fiber alignment), the manufacture and the reproducibility. They do not involve the use of toxic solvents and allow production on an industrial scale through spinning, weaving, knitting, non-woven and braided technologies [175,176]. Afterwards, a textile finishing can be applied to adjust, or determine, certain characteristics of the textile item: a fabric can be bleached or sterilized for medical use; a surface can be treated to become hydrophilic or superhydrophobic, depending on whether moisture absorption or repellency is required by the particular application; in some cases, like wound dressings, the two sides of the fabric may be tuned to behave differently; and a textile may be impregnated/coated with an agent(s) to confer specific properties, or to assist in the uptake or retention properties of the active agent [176].

Different strategies can be used to incorporate plant-extract-loaded particles into polymer-based solutions to extrude fibers, either by direct (e.g., co-axial spinning) or indirect (e.g., co-spinning) encapsulation [169]. Additionally, and alternatively, particles may be immobilized after obtaining the fibers, via entrapment between the fiber yarns and/or physical/chemical attachment to the fibers [177]. The immobilization of plant-extract-loaded CS-based organic particles onto fibers, fibrous assemblies, and textile fabrics can occur via three main types of chemical bonds, similarly as described elsewhere for the case of biomolecule’s immobilization onto natural fibers [54]: (a) physical adsorption, (b) physical entrapment, and (c) covalent bonding.

(a) Physical adsorption includes self-assembly methods such as van der Waals interactions, electrostatic interactions, hydrophobic effects, and affinity recognition [152,153];
(b) Physical entrapment of the particles within the fabric’s fibrous structure takes place either by vacuum induction or assisted by intermediary adhesive layers [154,155,156,157];
(c) Covalent bonding comprises short-range intermolecular attractive forces at the molecular scale [158,159,160].

There are frequently encountered combinations between the latter approaches, as well. On the other hand, the immobilization method trends of the aforementioned particles include the dip-pad-dry-cure method, impregnation, exhaustion method, spray-drying, and covalent chemistry. The goal is to immobilize a sufficient number of compounds in the fibrous templates, giving them enough bioactivity, for the necessary time period [54,161].

Fibers and textiles have been widely exploited as pharmaceutical repositories (e.g., drug-loaded carriers for medical therapy, nucleic acid delivery for gene therapy, enzyme carriers for biomedical applications), components of wound dressings (e.g., gauze, foam, hydrogels, transparent films, alginates, hydrocolloids, and antimicrobial dressings) and anti-adhesion membranes, percutaneous access devices, implantable devices (e.g., vascular stents and grafts, sewing rings, hernia repair meshes, skin/bone/cartilage/ligament mimetic scaffolds, nervous conduits, as well as drug delivery systems), sensors, reinforcing fillers, sound absorption, filtration systems, electrodialysis separation, or as part of personal protective equipment (PPE) and clothing (e.g., masks, surgical gowns, aprons, gloves, clothing, and hospital linen) [169,175,176,178]. The so-called biotextiles, based on natural and synthetic fibers, are defined as “structures composed of textile fibers and designed for uses in a specific biological environment where their performance depends on their interactions with cells and biological fluids as measured in terms of biocompatibility and biostability” [169,175]. These functional textiles are therefore designed and produced for their technical properties and performances, besides apparel and aesthetic purposes [179,180].

While facing all the above cited strategies and taking into consideration the aforementioned content of CS or plant extracts alone and the combination of CS as carrier skeleton and plant extract as payload, the integration of plant-extract-loaded CS-based organic particles into fiber-based systems gathers great potential for biomedical applications. Table 4 presents a comprehensive representation of existing studies on this subject.

Co-spun poly(ε−caprolactone) (PCL)/CS/curcumin nanofibers were fabricated by electrospinning, yielding nanofibers of ≈100 nm in diameter [172]. Then, curcumin-loaded electrostatically self-assembled CS/TPP NPs (via ionic gelation creating round-shaped NPs, with d ≈ 32 nm) were electrosprayed onto the surface of the previously prepared nanofibers, to improve the sustained release of curcumin at the wound site. Indeed, loaded nanofibers exhibited appropriate tensile mechanical properties, swelling behavior and water vapor transmission rate for use as wound dressing. In vitro testing revealed adequate degradation rate, curcumin release profile (22% in 6 h, 49% in 72 h), antioxidant potency, antibacterial efficiency (99.3 and 98.9% growth inhibition of MRSA and extended spectrum β-lactamase producing *E. coli* after 48 h), further allowing survival and proliferation of human dermal fibroblasts. In vivo studies showed 96.25% and 98.5% murine wound closure in 15 days, with and without MRSA infection. Bacterial growth inhibition was clearly perceived, enabling an enhanced reparative process of the skin, with well-organized connective tissue formation devoid of inflammation. Another example also resorted to curcumin-loaded CS/TPP NPs, this time freeze-dried and then dispersed into a PCL/gelatin (GN) solution prior to co-spinning [181]. This led to homogeneously distributed NPs of 359 nm of diameter and −10.7 mV of ζ potential and nanofibers with 1548 nm of diameter and suitable porosity (65%). Mats were endowed with good mechanical strength to act as a wound dressing material, in addition to a high swelling capacity, degradation profile, sustained drug release (23% in 6 h, 100% in 106 h), cytocompatibility towards human endometrial stem cells (favorable cell adhesion, proliferation and metabolic activity), and wound healing (82% wound closure at day 14). New tissue resembled normal skin, a regenerated wound, with clear re-epithelialization, normal rete ridges, and rejuvenation of skin appendages. Adding the stem cells to the mixture further decreased inflammatory signals and promoted angiogenesis. These studies show that dressings are in fact an essential part of the healing process, protecting the wound and intervening actively in the healing process [182].

Coaxial electrospinning was the strategy selected elsewhere [183] with PCL as core and polyvinylpyrrolidone (PVP)/veratric-acid-loaded CS/TPP NPs in the outer layer, to create nanofibrous mats encouraging bone tissue formation. Spherical NPs, with diameters ranging from 99 to 107 nm (and ζ potential of 16–18 mV), were found to increase nanofiber’s diameter up to ≈515 nm having clearly defined outlines, plus displaying commendable swelling and degradation behavior, mechanical properties, biomineralization efficiency, and in vitro drug release capacity (≈60% in 20 days). In vitro cell culture studies with mouse mesenchymal stem/stromal cells (mMSCs) resulted in valuable cytocompatibility, and osteoblastic differentiation potential (proven by alizarin red and von Kossa staining after 14 days of incubation, and gene expression levels of *RUNX2*, *ALPL*, *COL1A1,* and *BGLAP* at day 7 of contact with the biomaterial’s constructs). A different study, also aiming at bone regeneration, showed co-spun sinapic-acid-loaded CS/TPP NPs with PCL solution, which were also comprehensively characterized, and yielded exciting results in vitro with mMSCs and in an in vivo rat model including a critical-sized calvarial bone defect [184]. The cytocompatible constructs encouraged staining of alkaline phosphatase and calcium phosphate deposits, osteogenic potential at the gene and protein levels (runx2, type I collagen and osteocalcin) through probable activation of the TGF-β1/BMP/Runx2 signaling pathway. Micro-computed tomography imaging revealed significant new bone formation with the optimized constructs at 4 weeks, while histological staining (hematoxylin-eosin and Masson’s trichrome staining protocols) corroborated the later findings, leaving no doubt regarding their potential for bone neotissue formation. The self-healing capacity of bone is widely used to recover from small tissue injuries. However, bone grafts are needed to provide support, fill lacunae, and enhance biological repair/regeneration when the skeletal defect reaches a critical size [185].

Plant extract-loaded NP-mediated functionalization has also been widely applied to textile fabrics, with the main goal of obtaining prolonged biological effects [186]. One example explored citronella oil encapsulated within CS-based emulsions (having 79–93 nm in size on the course of 14 days of storage) further stabilized with citric acid onto a cotton knit (100% cotton textile, pore surface area of 20–90 µm^2^, distances within interyarn: 10–70 µm, distances within interfiber (intrayarn): 2–6 µm, and distances within pore (interfiber): 10–90 nm). Prior to particle immobilization through impregnation, the fabric was carefully washed and degassed by negative pressure. The results showed that nanocapsules were able to infiltrate the spacing of cotton textile fibers, including the fiber pores, thus leading to high washing durability (29% retention after 10 washing cycles). Others [187] included lemongrass oil loaded into emulsified CS NPs further physically-crosslinked with TPP (d ≈ 90 nm), which were then imprisoned into cotton fabric (plain-weave 112 g/m^2^, 60 ends per inch × 58 picks per inch) via dip-dry-curing while aimed at developing a durable anti-mosquito textile finishing. Acrylate was added as fabric adhesive to retain the nanogels adhered to the textile fibers. The roughness on fabric groves was maintained on the surface after dry and wet crocking, with the artificial sweat (acidic and alkaline) treated fabrics also retaining the roughness due to the presence of the capsules. Efficacy was proven even after 15 washing cycles, still enabling 75% of repellency against mosquitoes following the activity of the entrapped compounds (100% bioactivity without washing). Additionally, given that 36 days of repeated application of nanogel on mice’s skin was nontoxic, the tested formulation was found to be suitable as protective clothing of military personnel and individuals at risk for mosquito bites in the line of duty. Of particular note is the extra caution that these recently published studies had with fabric pre-immobilization procedures, particularly in appropriately washing it so that no contaminants compromised the intended subsequent bond formations nor the fabric’s applicability. Finally, one last example [188] emphasizes the value of functional and antimicrobial textile fabrics by immobilizing such herbal nanotechnologies, given that nearly half of the medical textiles dwell around antimicrobial treatments [176]. A pad-drying approach was used to coat the cotton fibers with the plant-derived (*Aloe vera* nano-sized powder) CS NPs (d ≈ 40 nm), leading to a conglomerate with UV-blocking properties (while exhibiting absorbance in the UV region at 269 nm, and having UPF > 50), antibacterial activity (ZoIs of ≈27 mm and ≈22 against *S. aureus* and *E. coli*, respectively, being close to the results obtained with the antibiotic amoxicillin (ZoIs of ≈28 mm and ≈23)), and superhydrophobicity (≈155°), even after 10 washes.

**Table 4:** Integrative strategies of plant extract-loaded CS-based small-scaled organic particles onto fiber-based systems for biomedical applications.

Fiber-Based Structure			Immobilization Strategy	Loaded Carrier			Main Chemical Bonds between Carrier and Fiber	Bioactivity	Potential Application	Ref.
Materials	Processing	Functional Groups		Composition	Plant Extract	Preparation Method				
Collagen/fibrin	Cryodesiccation	-	Dispersion (solubilization until NP homogenization was reached within the polymeric solution)	CS/TPP	Gallic acid	Ionic gelation	-	At 3, 6, 24, and 72 h, 9.71 ± 2.3%, 20.69 ± 3.9%, ≈41% and ≈72% of gallic acid was released from the scaffolds. The engineered scaffold accelerated angiogenesis, hexosamine synthesis, collagen deposition and recruited immune cells at wound area.	Wound healing	[104]
PCL/CS/Curcumin	Electrospinning	-	Electrospraying PCL/CS/curcumin nanofibers with curcumin-loaded CS NPs	CS/TPP	Curcumin	Ionic gelation	-	Improved antibacterial, antioxidant, and cell proliferation efficiencies, with higher swelling capability and water vapor transition rate of the electrospun fibers. In vivo examination showed significant improvement of wound healing in MRSA-infected wounds.	Wound healing	[172]
PCL/GN	Electrospinning	-	Dispersion (solubilization until NP homogenization was reached within the polymeric solution)	CS/TPP	Curcumin	Ionic gelation	-	Improved biocompatibility and wound healing abilities in a full-thickness excisional animal model. Cell attachment and proliferation was enhanced in the presence of the NPs.	Wound healing and skin substitutes	[181]
PCL/PVP	Co-axial electrospinning (sheath PCL and core PVP)	-	Solubilization of the NPs with PVP portion of the fiber and extrusion as the core of the electrospun fibers	CS/TPP	Veratric acid	Ionic gelation	-	Reached 60% release after 20 days of incubation. Modified fibers were biocompatible with mouse mesenchymal stem cells, promoting their differentiation (upregulation of bone differentiation-related markers).	Bone regeneration	[183]
PCL	Electrospinning	-	Dispersion (solubilization until NP homogenization was reached within the polymeric solution)	CS/TPP	Sinapic acid	Ionic gelation	-	Enhanced osteoblast differentiation and activated the osteogenesis signaling pathways in mouse mesenchymal stem cells. In vivo data reflected the extract ability to instigate bone formation.	Bone regeneration	[184]
Wool	-	-OH	Pad-dry-cure technique	CS/TPP	Propolis	Ionic gelation	Hydrogen bonding and physisorption.	Enhanced antimicrobial action against fungi and bacteria. Synergistic effects with textile dyes (improved antimicrobial protection).	Textile finishes for microbial-protective clothing	[189]
Cotton	Dip in 3% NaOH for 45 min, soaked in 10 g·L−1 sodium dodecyl sulphate for 30 min and in hot ethanol for 30 min. Then, washed with boiling ultrapure water for 5 times and dried at 25 °C under 65% relative humidity for use. Prior to surface modification, fabric degassed by negative pressure	-OH	Immersion in particle dispersion at 40 °C, 100 rpm/min for 1 h. Wet pick up of 100%. The finished textile was dipped into deionized water and placed into constant temperature and pressure to dry the textile and remove the extra water.	CS, citric acid, CO-40, TGI or CS, citric acid, Span 80, Tween 80	Citronella oil	Emulsification and ionic gelation	Hydrogen bonding between particles and textile fibers, and electrostatic interaction with -NH_2_ of CS	Aromatic retention of 28.84% after 10 washing cycles.	Aromatic textile finishing	[186]
Cotton	Non-ionic detergent used at 25 °C for 30 min for fabric washing, warm water then cold water applied, and finally, fabric drying	-OH	Dip-dry-cure: Immersion in 100 g/L of gel on a shaker at 1000 rpm at 25 °C for 2 h, dried at 50 °C for 5 min and cured at 100 °C for 2 min, rinsing with water to remove unbound or loosely bounded molecules.	CS, Tween 80, TPP/acrylate	Lemongrass oil	Emulsification followed by ionic gelation. Acrylate added as fabric adhesive	Hydrogen bonding between particles and textile fibers, and electrostatic interaction with -NH_2_ of CS	100% of repellency against mosquitoes (75% after 15 washes). Absence of dermal toxicity in mice.	Insect-repellent clothing	[187]
Cotton	Perfumed cotton fabrics initially washed with water at 40 °C, drained and rinsed with water at 25 °C and finally spun.	-OH	Impregnation: immersion in particle dispersion for 2 h under vacuum (100 Pa) at 30 °C, air-drying at 50 °C with the air current rate of 0.4 m/s for 1 h in the oven (moisture content: 0.01103 kg/m^3^).	CS, Tween 80/TPP	Rose fragrance	Emulsification followed by ionic gelation	Hydrogen bonding between particles and textile fibers, and electrostatic interaction with -NH_2_ of CS	80% plant extract release in 20 washing cycles. 55% release in 10 days at 70 °C, 0.4 m/s of air current rate and moisture content of 0.01 kg/m^3^.	Long-term fragrance-releasing textiles	[190]
Cotton	-	-OH	Dip-pad-dry-cure method, with fabric immersed in carrier dispersion and citric acid binder (1%) for 5 min, padded 15 m/min with a pressure of 1 kgf/cm^2^, air-dried, cured 3 min at 140 °C and immersed 5 min in sodium lauryl sulfate to remove unbound NPs and the soap solution, followed by air-drying.	CS, Tween 80, Span 80, palm oil and TPP	Neem methanolic extract	Emulsification followed by ionic gelation	Esterification with -COOH of citric acid also promoting electrostatic interaction with -NH_2_ of CS	Enhanced antibacterial efficiency (until 20 laundry washes): 100% *S. aureus* reduction (ZoI: 20 mm) and 93% *E. coli* reduction (ZoI: 14 mm).	Textile finishes for bacterial protective clothing	[180]
Cotton	-	-OH	Dip-pad-dry-cure: immersion in particle dispersion and citric acid binder for 5 min, padding mangle to remove excess solution, with 100% wet pick-up, air-drying, curing at 140 °C for 3 min, immersion in sodium lauryl sulfate for 5 min to remove unbound extract, rinsing to remove the soap solution and air-drying.	Alginate, CaCl_2_, CS	Methanol extracts of *Ocimum sanctum*	Ionic gelation and polyelectrolyte complexation	Esterification with -COOH of citric acid also promoting electrostatic interaction with -NH_2_ of CS	100% (*B. cereus*, *P. aeruginosa,* and *S. aureus*) or 98% (*E. coli*) bacterial reduction, effective until 20 or 10 washing cycles.	Biocontrol agent against bacteria in fabrics	[191]
Cotton	Fabric washed 0, 5, 10, 15, and 25 times, washing with 2% soapy water for 15 min, and rinsing in clean water	-OH	Dip-dry-cure: immersion in bath containing microcapsule emulsion, 2D resin, catalytic agent, and JFC penetrant. Wet pick up at 100%, drying at 80 °C for 3 min, curing at 160 °C for 2 min, and then cooling down to room temperature. Washing and drying.	CS, gelatin, span-80, glutaraldehyde	Patchouli oil	Emulsification and chemical crosslinking	Crosslinking between 2D resin and hydroxyl groups of cotton and/or microcapsules through acid-catalyzed dehydration	Gradual decrease of antibacterial activity down to 75 and 70% (against *S. aureus* and *E. coli, respectively*) after 25 washes.	Antibacterial mask, bacteriostatic sheet and health-care clothes	[192]
Cotton	Textile binder (Knittex CHN, melamine resin) used to enhance microcapsule fixation to the fabric	-NH_2_	Dip-pad-dry-cure: immersion in microcapsule solution, vertical padding 1.5 kg/cm^2^ and 7.5 rpm with two dips and two nips, drying at 80 °C for 3 min, curing in a Mathis curing oven at 100 °C for 3 min, and air-drying.	CS, alginate, liquid paraffin, Span 80, NaOH, glutaraldehyde	PentaHerbs aqueous extracts	Polyelectrolyte complexation, emulsification, and chemical crosslinking	Electrostatic interaction of -NH_2_ of melamine resin and -COOH of alginate	Cytocompatible towards human epidermal equivalent.	Garment development for atopic dermatitis	[193]
Cotton	-	-OH	Dip-pad-dry-cure: immersion in microcapsule dispersion, sodium hypophosphite (catalyst), citric acid, and deionized water (bath ratio = 1:20) for 70 min; rolling (two dips and two rollings; wet pick up, 80%; pressure, 0.3 MPa). Drying at 90 °C for 3 min, curing at 160 °C for 2 min, then cooling to room temperature. Washing with water and drying under vacuum at 60 °C for 24 h.	CS, citric acid	Vanillin ethanolic solution	Emulsification and ionic gelation	Esterification with -COOH of citric acid also promoting electrostatic interaction with -NH_2_ of CS	Sustained drug release until 14 laundry washes.	Functional fibers in the textile industry	[194]
Cellulose	Fibers washed with 1% non-ionic detergent at 30 °C for 30 min and rinsed with water for 15 min	-OH	Dip-pad-dry: immersion in particle dispersion, padding at 2.5 m/min and 4 bars to remove excess solution, air-drying, rinsing with deionized water, and air-drying again.	CS, surfactant, NaOH	Limonene oil	Emulsification and neutralization	Hydrogen bonding between particles and textile fibers, and electrostatic interaction with -NH_2_ of CS	Decreased oil volatility in 8 h.	Insect repellent for textiles	[195]
Cotton	-	-OH	Pad-dry: padding at 35 rpm for 5 min and drying at 60 °C for 10 min	CS	Aloe vera herbal nanopowder	Coating	Hydrogen bonding between particles and textile fibers, and electrostatic interaction with -NH_2_ of CS	ZoI of 22 mm and 27 mm against *E. coli* and *S. aureus*, respectively, UV-protection factor of 57 and superhydrophobicity 155°.	Antibacterial protective clothing	[188]

## 5. Conclusions and Future Perspectives

In the biomedical field, recent studies (between 2020 and 2021) mostly exploit CS and plant extracts forming nanocomposites, directed at infection prevention or control, additionally acting against oxidative stress and inflammation overload, which are critical for instance in immunocompromised patients [196,197,198,199,200,201,202,203]. CS is a marine-derived cationic polysaccharide, approved by the FDA (Food and Drug Administration) for wound dressing applications and cartilage repairing formulations. It has been approved as functional food in some Asian countries, recognized as safe (GRAS) and approved for dietary use in Italy and Finland [177]. Its biocompatibility, bioactivity, chemical versatility, and ease of processing into a variety of structures make it a strong contender for use in numerous biomedical applications, including microbicidal approaches. As communicable diseases threaten to reach epidemic proportions, affecting patients from all ages, societal and financial status, with high mortality rates and healthcare burden, the search for effective antimicrobial strategies is highly needed. In parallel, plant extracts (widely used as folk medicine) are increasingly considered as potential alternatives to antibiotics, having high potential for being a source of natural drugs that can be used to counteract microbial survival and prosperity [22,54,55]. Several natural drugs have already been approved for clinical use, namely for antioxidant and antimicrobial purposes [178,179]. Notwithstanding, their combination as organic particles has also been object of several studies, while emphasizing their antioxidant [158,204,205] and antimicrobial capabilities [204,206,207]. If loaded into CS-based nano- or micro-scale organic particles [115], plant extracts hold the power to confer strong and long-lasting effects, without the consequences of overdose-induced tissue damage or inefficiency due to drug biodegradation ahead of reaching the target site. Their integration into suitable carriers can protect the drug from biodegradation, transport it into the target site, enable a controlled release and avoid off-target action, thus heightening, or complementing, their biological effects [2,180,181]. CS-based small-scale organic carriers have been widely studied for biomedical applications, having tremendous potential [2,182,183]. However, the development of plant-extract-loaded formulations is still limited, namely when applied to fiber-based systems. Nowadays, electrospinning is the most frequently studied technique to produce fibers (at the nanoscale) carrying these NPs, namely via co-spinning. The compositions are being mainly sought out for wound dressing layers additionally encouraging tissue regeneration. Large bone lesions may also benefit from such strategies. On the other hand, cotton is currently the sole type of fabric being functionalized with these types of particles, with no major pre-treatment being applied. Particle addition is mostly done by dip-pad-dry-cure method, frequently with citric acid acting as linker between CS and the cotton fibers, and the particles being produced via emulsification followed by ionic gelation methods encapsulating the hydrophobic plant extracts. An antimicrobial finishing has been the major added functionality. However, poor mechanical properties, washing durability, and burst release are still frequently encountered obstacles. Yet, research is progressing very well, with the production of numerous and clever CS derivatives, added processing methodologies, crosslinking steps, and functionalization protocols, thus creating high hopes to overcome these limitations and stick to its beneficial traits. Moreover, triggers such as ionic strength, temperature shifts, enzymatic reactions, oxidative stress, or even light irradiation, not as explored in this context, could also lead to thrilling new avenues of plant-derived drug consumption under the appropriate pathological settings. These scientific advancements can be of great utility to produce safe and effective bioactive medical textiles. Scaffolding systems, coatings, wound dressings, sutures, face masks, and hospital linen are a few relevant examples.

## Figures and Tables

**Table 1:** Latest trends exploring the antibacterial capacity of CS or CS derivatives while integrating different processed architectures, including main attributes of the created polymer (DA and Mw) or its derivatives (name of the derivative, DA, degree of substitution (DS) and Mw), biomaterial-processed structures, afflicted bacteria, and intended application.

CS or CS Derivative				CS-Based Structure(s)	AM Features	Afflicted Bacteria	Intended Application	Ref.
DA	Derivative	DS	Mw					
23–62%	Thymine-modified CS	-	154–194 kDa	CS porous sponges	Wrinkled and damaged cell walls, particularly with increased DS, which increased CS’ solubility and charge density. 100% cell death.	*Staphylococcus aureus,* methicillin-resistant *Staphylococcus aureus* (MRSA)*, Escherichia coli, Pseudomonas aeruginosa,* and *Acinetobacter baumannii*	Wound dressing	[31]
-	-	-	50–190 kDa	Core [gelatin (GN) + poly(vinylpolypyrrolidone) (PVP) + imipenem/cilastatin]—shell (CS + poly(ethylene oxide) (PEO) + vancomycin) nanofibers	Zone of inhibition (ZoI) of 2.45, 2.90, 2.75, and 1.85 cm, respectively. CS enabled controlled release of the antibody for higher global efficiency.	*MRSA, S. aureus, P. Aeruginosa,* and *E. coli*	Wound dressing	[32]
≥10%	Carboxymethyl CS	≥20%	10–20 kDa	Carboxymethyl CS loaded with waterborne polyurethane–GN hydrolysate hydrogel film	ZoI of 12–16 and 16–20 mm, respectively. Higher activity of higher CS derivative amount, especially at lower pH.	*S. aureus* and *E. coli*	Wound dressing	[33]
9.7%	-	-	100–300 kDa	Cinnamon leaf or clove-oil-loaded CS and poly(vinyl alcohol) (PVA) blended films	CS films alone were effective against both bacteria and capable of eradicating all *P. aeruginosa* in 1 h (*** *p* < 0.001). Still, loaded CS/PVA films showed significantly improved AM traits in relation to unloaded films within 2 h of contact.	*S. aureus* and *P. aeruginosa*	Wound dressing	[34]
15%	-	-	Low	Thyme-oil-loaded CS-tripolyphosphate (TPP) microcapsules spray dried onto linen fabric	>98% growth inhibition due to oil and CS joint action.	*E. coli*	Textile finishing	[35]
26%	-	-	292 kDa	TiO_2_ nanoparticles (NPs) dispersed onto CS–glycerol-coated cotton fabric	99.8 and 97.3% bacterial reduction, respectively, driven by CS’s cationic nature.	*S. aureus* and *E. coli*	Textile finishing	[36]
15%	Quaternized tosyl CS	45–55%	-	Crosslinked hydrogels of CS derivative and GN	Quaternary CS (replacing primary -OH) and free amino groups interacted with the anionic bacterial membrane, and the lipophilic chain perturbed the hydrophobic domains of the cell envelope. Minimum inhibitory concentration (MIC): 128−256, 64−128, 256, 256−512, 64 to 128, 64−128, 64−256, and 256−512 μg/mL, respectively.	MRSA, *S. epidermidis*, *P. aeruginosa, A. baumannii*, vancomycin-resistant *S. aureus* (VRSA), *E. faecium*, vancomycin-resistant *Enterococcus* (VRE) and *E. coli*	Healthcare infection control	[37]
15%	-	-	Medium	TPP-crosslinked CS, GN, potato-starch, and banana peel powder (BPP) blended films	ZoI of 5–8 (*S. aureus*) and 9–11 mm (*E. coli*), because of the CS and BPP combined effect.	*S. aureus* and *E. coli*	Wound dressings	[38]
29%	-	-	-	CS-coated UV-disinfected Vicryl sutures	Growth inhibition of both bacterial and fungal pathogens, profound inhibition of slime formation, and mixed-species biofilm inhibition, as a result of CS’s activity. No hyphal formation.	*S. epidermidis* and *Candida albicans*	Surgical sutures	[39]
10%	O-carboxymethyl CS	80%	200 kDa	O-carboxymethyl CS and Jeffamine porous hydrogel	≥99% bacterial reduction on account of CS’s amine groups.	*E. coli*	Wound dressing, drug delivery, and tissue engineering	[40]
-	Methacrylated glycol CS	70%	-	β-cyclodextrin-/triclosan-complex-grafted methacrylated glycol CS	Full inhibition of bacterial infection in 5 h and improved wound healing, attributed to the hydrophilic/hydrophobic nature of CS derivative.	*S. aureus* and *E. coli*	Tissue adhesives for wound closure	[41]
-	Quaternized CS	26%	-	Crosslinked (carbodiimide chemistry) quaternized CS-coated titanium printed cages	ZoI: 15 mm^2^, 0 CFU/mL, decreased crystal violet staining, in vivo inhibition of bacterial growth throughout the entire observation period (1–5 d), and reduced bacterial quantity in the extracted cages.	*S. aureus*	Intervertebral fusion cages	[42]
15%	-	-	5–20 mPa.s	Spray-dried CBO-loaded CS and gelatin microcapsules	Over 90% growth inhibition until 10 fabric washes.	*S. aureus* and *E. coli*	Functional finishing of linen	[43]
-	-	-	Medium	Self-assembled nanogels of gluthathione–silver (Ag) nanoclusters (NCs) and CS	Improved antibacterial action (>10-fold), with the well-dispersion of the ultrasmall Ag NCs in the CS framework protecting Ag NCs from decomposition and aggregation and allowing a slow release of Ag^+^ ions; the positively charged CS carrier substantially promotes Ag–bacteria interaction and the concomitant Ag bactericidal activity.	*S. aureus, E. coli, Bacillus subtilis,* and *P. aeruginosa*	Theranostic nanomedicines	[44]
21%	-	-	Medium	CS-TPP NPs incorporated within cotton fabric via pad-dry-curing	Increased ZoI: ≈20 (*S. aureus* and *B. subtilis*), ≈16 (*E. coli* and *Proteus vulgaris*), ≈25 mm (*C. albicans* and *A. Niger*) due to CS-based NP action.	*S. aureus, B. subtilis, E. coli, Proteus vulgaris, C. albicans* and *Aspergillus Niger*	Textile finishing	[45]
-	-	-	-	CS-coated PCl microparticles (MPs) encapsulating Ag NPs, then entrapped into PVA/PVP microneedle layers	pH-triggered Ag release enabled 100% eradication of bacterial bioburdens from an ex vivo biofilm model in rat skin, given the feasibility of the loading of silver NPs into responsive MPs.	*S. aureus* and *P. aeruginosa*	Biofilm skin infections	[46]
-	-	-	-	CS hydrogel combined with zinc oxide/zeolite nanocomposite	33 and 45% biofilm formation and metabolic activity reduction, due to a joint effect from the nanocomposite’s elements. Significantly decreased gtfB, gtfC, and ftf reinforcing lower bacterial adhesion.	*Streptococcus mutans*	Dental biofilm control	[47]
-	Double bond modified N-dodecylated CS	-	-	Macroporous cryogel containing double bond modified N-dodecylated CS and graphene oxide (GO)	Excellent near-infrared (NIR)-assisted photothermal antibacterial activity against both bacteria and killed 99% of them after 20 min NIR irradiation, because of the CS derivative and GO.	*S. aureus* and *E. coli*	Clinical hemorrhage and infection control	[48]
5–10%	Quaternary CS	76.4%	340 kDa	Quaternary CS/PVA nanofiber membrane crosslinked with blocked diisocyanate	~100% antibacterial efficacy, attributed to the CS derivative permanently cationic net charge.	*E. coli*	Wound dressings	[49]
<25%	-	-	310–375 kDa	Graphene/CS/magnetite NPs	ZoI of 21.3 and 19.3 mm and MIC of 60 and 70 μg/mL, respectively, due to synergistic antibacterial action of NP constituents.	*ESBL-producing P. aeruginosa* and *Klebsiella pneumoniae*	Biomedical applications with antibacterial requirement	[50]
-	N–succinyl CS	-	150 kDa	PVA/N–succinyl CS/lincomycin porous hydrogels	~100% and ~70% antibacterial efficacy, respectively, with the antibiotic being held responsible for most of it.	*S. aureus* and *E. coli*	Wound dressings	[51]
15–25%	-	-	Medium	Ag NP-doped multilayered CS hydrogel	ZoI: ~7 and ~12 mm, promoted by staged release pattern of Ag NPs based on acid triggered dissolution of the multi-membrane layer by layer.	*S. aureus* and *E. coli*	Implant coating or wound dressings	[52]
-	Quaternized CS	-	-	Double-crosslinked oxidized dextran–dopamine and quaternary CS with encapsulated Ag NPs and deferoxamine	15.5 and 20.8% survival rate, plus 3/97% and 9/91% live/dead cells, respectively, through the combination of Ag NPs and HTCC.	*S. aureus* and *E. coli*	Bacterial infected diabetic wound dressing	[53]
-	Quaternized CS	22%	-	Protocatechuic-acid-grafted quaternized CS	Excellent antibacterial properties and showed a satisfactory synergistic antibacterial effect with protocatechuic acid.	*S. aureus* and *MRSA*	Infection control	[54]
15–25%	-	-	50–190 kDa	Tea-tree-oil-loaded CS-poly(ε-caprolactone) core-shell nanocapsules	Increased cell death (17%), following contact with released essential oil and CS shell.	*Cutibacterium acnes*	Topical acne treatment	[55]
-	-	-	-	Thiolated CS/Ag nanowire composite hydrogels	Increased ZoI because of CS derivative and Ag joint action.	*S. aureus* and *E. coli*	Obstetric wound care	[56]
8%	Fluorinated quaternary CS	-	50–190 kDa	Fluorinated quaternary CS	Bacterial cell death in 6 h. MICs of 64 to 512 μg/mL (Gram-positive bacteria) and 128 to 512 (Gram-negative bacteria), particularly effective against MRSA and *B. subtilis*. Fluorination and quaternization of CS improved its solubility and antimicrobial activity. Fluorine is the most electronegative element with a strong effect on the conformational and physicochemical properties of organic compounds.	MRSA, *E. coli, P. aeruginosa, Streptococcus sanguinis, Salmonella enterica, S. epidermidis, B. subtilis,* and *S. aureus*	Infection control	[57]
15–25%	Mannose-functionalized CS	-	Medium	Mannose-functionalized CS nanosystems	Particular bacterial growth inhibition (4× lower), anti-adherence (4× lower), and biofilm disruption (3–6× lower) activity. Electrostatic interaction disturbed the bacterial membrane integrity, osmolarity, and depletion of nutrients. With mannose, it interacted with bacterial membrane lectins, interfering with adhesion and motility.	Multidrug-resistant clinical isolates of *E. coli, Listeria monocytogenes, S. aureus,* and *P. aeruginosa*	Infection control	[58]
15%	N-halamine hydantoin-containing CS	56%	250 kDa	N-halamine hydantoin-containing CS films	0.003% and 0.218% CFU/mL, on account of the biocidal N–Cl bonds added to the already antibacterial CS.	*S. aureus* and *E. coli*	Infection control	[59]
15–25%	-	-	100–300 kDa	CS–hyaluronic acid polyelectrolyte multilayered coating of nylon monofilament sutures	Significant growth inhibition in the first hours of contact, given antibacterial features of the built coating.	*S. aureus* and *E. coli*	Sutures	[60]
15–25%	Catechol-modified quaternized CS		Medium	Catechol modified quaternized CS incorporated into PDLLA-PEG-PDLLA hydrogel	>95% bacterial cell death, potentiated by the quaternized CS moieties.	*S. aureus* and *E. coli*	Wound dressings	[61]
-	-	-	-	Cellulose acetate nanofibers coated with CS nanowhiskers	99% growth inhibition due to CS nanowhisker activity.	*E. coli*	Biomedical applications with antibacterial requirement	[62]
-	N-succinyl CS	-	Low	N-succinyl CS-ZnO NPs conjugated with curcumin	MIC reduction of 25-to-50-fold and minimum bactericidal concentration (MBC) reduction of 10-to-40-fold, respectively, given curcumin addition to NPs containing CS derivative and ZnO, all endowed with antibacterial traits.	*S. aureus* and *E. coli*	Biomedical applications with antibacterial requirement	[63]
-	-	-	Medium	CS and β-glycerolphosphate hydrogel	In vitro unresponsiveness but clear in vivo bacterial reduction, as treated wounds were completely re-epithelialized and closed on day 14 post-surgery.	*A. baumannii*	Wound dressings	[64]

**Table 2:** Main classes of antibacterial plant constituents based on the division proposed by Cowan [69], in addition to representative chemical structures of relevant examples.

Antibacterial Compound Classes		Description	Examples		Ref.
Phenols and polyphenols	Simple phenols	Single substituted phenolic ring	Eugenol 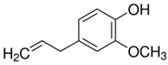	Thymol 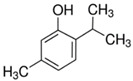	[34,54,70,87,91,92,93]
	Phenolic acids	C6-C1 (hydroxybenzoic acids) or C6-C3 (hydroxycinnamic acids), consisting of a phenolic ring and a carboxyl substituent	Gallic acid 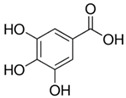	Protocatechuic acid 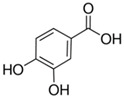	[34,54,70,87,91,92,93]
	Quinones	Aromatic rings with two carbonyl groups	Thymoquinone 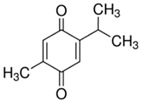	1,4-Naphthoquinone 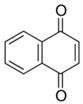	[94,95,96,97,98,99,100,101]
	Flavonoids	Phenolic compounds that include a C6-C3-C6 carbon framework (phenyl benzopyran)	Quercetin 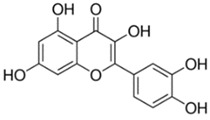	Vaccarin 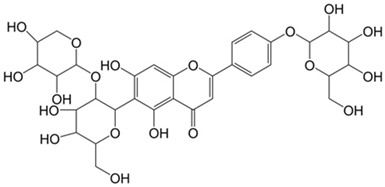	[102,103,104,105,106,107]
	Tannins	Hydrolysable tannins: central core of glucose or another polyol esterified with gallic acid, also called gallotannins, or with hexahydroxydiphenic acid, also called ellagitannins; condensed tannins: oligomers or polymers composed of flavan-3-ol nuclei.	Gallocatechin 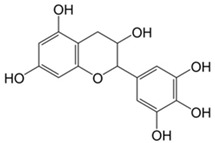	(−)-Epigallocatechin gallate 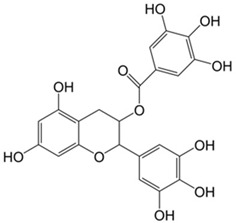	[14,108,109]
	Coumarins	Phenolic substances with fused benzene and α-pyrone rings	Novobiocin 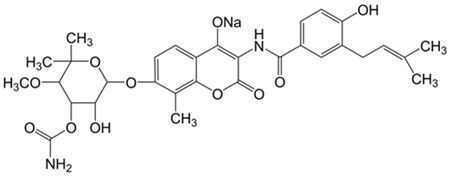	Chlorobiocin 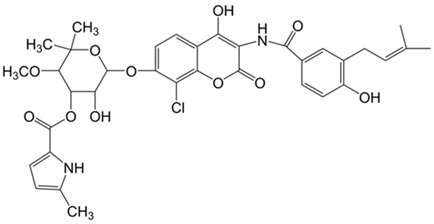	[77,78,79,80]
	Terpenes and terpenoids	General chemical structure is C_10_H_16_, and they occur as diterpenes, triterpenes, and tetraterpenes (C_20_, C_30_, and C_40_), hemiterpenes (C_5_) and sesquiterpenes (C_15_). In terpenoids methyl groups are moved/removed, or functional groups (usually oxygen-containing) are added.	Limonene 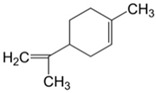	Geraniol 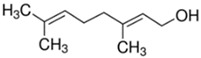	[81,110,111,112,113,114,115]
	Alkaloids	Heterocyclic nitrogen compounds	Tetrandrine 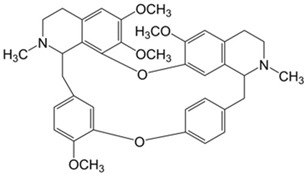	Berberine 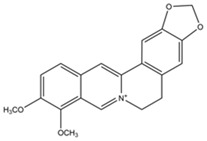	[116,117,118,119,120]

**Table 3:** CS-based small-scale organic particles loaded with plant extracts for biomedical applications.

CS or CS Derivative				Carrier Composition	Production Method	Loaded Plant Extract		Main Particle Features	Main Observed Effects	Potential Applications	Appointed Release Mechanism	Ref.
DA	Derivative	DS	Mw			Hydrophilic	Hydrophobic					
15	-	-	60 kDa	CS/poly(γ-glutamic acid) (γ-PGA)	Polyelectrolyte complexation	-	Tea catechins	Round-shaped; d_DLS_ = 134–147 nm; ζ = −18.7–33.5 mV (Δ molar ratio)	Enhanced antioxidant activity.	Dietary supplements	pH-triggered disintegration	[14]
-	-	-	Low	CS/TPP	Ionic gelation	-	Grape pomace extract	Round-shaped; d_DLS_ = 419–853 nm; ζ = 7.4–14.9 mV (Δ CS and drug concentration)	High antioxidant capacity and antimicrobial action against methicillin-susceptible *S. aureus*, *L. monocytogenes*, *P. aeruginosa*, *S. enteritidis*, *E. coli*, and *C. albicans*. Reduced in vitro intestinal permeability.	Dietary supplements	pH-triggered release	[157]
-	-	-	100–200 kDa	CS/TPP	Ionic gelation	P. dactylifera extract	-	Round-shaped; d_DLS_ ≈ 210 nm; ζ = 33 mV	Antioxidant, antibacterial, antifungal, and anticancer (yet protecting vital organs from oxidative stress).	Dietary supplements	-	[158]
15–25%	-	-	-	CS/lecithin	Nanoprecipitation or solvent displacement	-	Thyme	Round-shaped; d_TEM_ = 6.4 (NPs) or 9.1 nm (nanocapsules)	Controllable release kinetics with significant inhibitory effects against *S. aureus* and *Bacillus cereus*.	Antimicrobial medication against foodborne bacteria	Desorption, or diffusion	[88]
-	-	-	190–310 kDa	CS/fucoidan	Polyelectrolyte complexation	-	Quercetin	d_DLS_ = 356 nm; ζ = −30 mV	Controlled release under biorelevant simulated gastrointestinal environments; significant antioxidant activity.	Nutraceutical and pharmaceutical uses	pH-responsive diffusion, combined with carrier erosion	[159]
15–25%	-	-	Medium	CS/TPP/sodium hexametaphosphte (HMP)	Emulsification and ionic gelation	-	*Carum copticum*	Round-shaped; d_DLS_ = 236.0–721.0 nm	Improved antimicrobial and antioxidant effects.	Nutraceutical, cosmetic and pharmaceutical uses	Desorption, then diffusion, especially in alkaline conditions	[149]
15.2%	-	-	Medium	CS/TPP/Tween 80	Emulsification followed by ionic gelation	*-*	Peppermint and green tea oils	Round-shaped; d_TEM_ = 20–60 nm, d_DLS_ = 252.6–256.3 nm; ζ = −20.9–29.0 mV (Δ molar ratio)	Increased thermal stability; enhanced antioxidant activity and antimicrobial action.	Nutraceuticals, cosmetic and pharmaceutical uses.	Diffusion	[75]
-	-	-	Low	CS/TPP	Ionic gelation	-	*Physalis alkekengi-L*	Round-shaped; d_SEM_ = ~160 nm, d_DLS_ = 196 nm; ζ= 7.69 mV	Prolonged antioxidant activity with potential anticancer performance.	Antioxidant medical formulations	Diffusion	[160]
4%	-	-	Medium	CS/polysorbate 80	Ionic gelation	-	Thymoquinone	Round-shaped; d_TEM_ = 74.66 nm, d_DLS_ = 492.3 nm; ζ= 3.89 mV	Elevated monoamine neurotransmitter synthesis, particularly serotonin, and prevented oxidative stress on neural cells (enhanced antidepressant effects).	Antidepressants for mental illnesses	Desorption, then diffusion	[76]
14%	-	-	~50 kDa	CS/TPP	Ionic gelation	-	Rosmarinic acid, *Salvia officinalis* (sage) and *Satureja montana* (savory)	Round-shaped; d_DLS_ = 280.0–302.4 nm; ζ= 27.5–30.1 mV	Increased permeability and retention; no cytotoxic effects.	Treatment of oxidative eye conditions	pH-triggered disintegration	[161]
-	-	-	-	Carboxymethyl CS, hydroxypropyl CS or trimethyl CS/poloxamer 407/Kolliphor^®^ HS 15	Emulsification	-	Tetrandrine	Round-shaped; d_DLS_ = 157.0 nm; ζ = 22.1 mV	Improved drug sustained release and bioavailability; no sign of ocular irritation.	Treatment of glaucoma	Desorption, then diffusion	[119]
≤25%	-	-	-	CS/alginate/tween 80/CaCl_2_	Emulsification and ionic gelation	-	Turmeric and lemongrass oil	Round-shaped; d_DLS_ = 226.4–256.6 nm; ζ = 35.7–37.3 mV	Hemocompatible, nontoxic systems with a sustained drug release profile; antibacterial, antifungal, antioxidant, antimutagenic, and anticarcinogenic properties.	Medical and pharmaceutical drug delivery systems	pH-responsive diffusion	[73]
10	-	-	150 kDa	Citric acid-CS/TPP and N, N, N-trimethyl CS/TPP	Emulsification and ionic gelation	-	*Ocimum gratissimum* essential oil	Round-shaped; d_DLS_ = 134.9 and 153.5 nm; ζ = 26.1 and 22.6 mV, respectively	Increasing antioxidant activity even after 75 h. With, CS derivative, antibacterial activity at a lower concentration for both Gram-negative and Gram-positive food pathogens. Toxic towards MDA-MB-231 breast cancer cell lines.	Antioxidant, antibacterial and anticancer agents	Desorption, then diffusion	[151]
-	-	-	-	CS grafted to mesoporous silica NPs	Emulsification, chemical grafting and gate-penetration by super-critical CO_2_	-	Zedoary oil	Mesoporous round-shaped; d_DLS_ = 86.7 nm	Controlled release triggered by pH changes; increased stability of the loaded molecule	Drug delivery systems	pH-responsive diffusion	[162]
≤15%	-	-	Medium	CS/*Pterocarpus marsupium*	Ionic gelation	*Pterocarpus marsupium*	-	Round-shaped; d_DLS_ = 676 nm; ζ = 57.3 mV	Higher stability, enhanced entrapment efficiency, and sustained drug release characteristics. Significant increase in alpha-amylase inhibition and appreciable anti-inflammatory activity.	Therapeutic agent against diabetes and inflammatory disorders in drug delivery applications	Desorption, then slow degradation and diffusion	[163]
15%	-	-	50–190 kDa	CS/quinoline/Tween 60	Nanoemulsion	-	Quercetin	Nanorod shape and monolithic structure; d_DLS_ = 141–174.8 nm; ζ = −2.4 to −14.1 mV	Enhanced pH-sensitive controlled release; remarkable anticancer activity against HeLa cells by reducing cancer cells’ proliferative skills.	Anticancer drug nanocarriers	pH-responsive diffusion	[74]
15–25%	-	-	50–190 kDa	CS/TPP	Ionic gelation	-	*Posidonia oceanica*	Round-shaped; d_DLS_ = 252.4 nm; ζ= 19.7 mV	Excellent physical and chemical stability during storage; enhanced extract solubility and prolonged release; improved inhibitory effect on cell migration.	Treatments to prevent neuroblastoma cell migration	Diffusion	[164]
-	-	-	-	CS/Tween 20	Nanoemulsification	-	Zataria multiflora oil	Round-shaped; d_DLS_ = 463 nm; ζ = 18.35 mV	Improved the proliferation inhibition rate of breast cancer cells by inducing apoptosis, generating ROS, and triggering mitochondrial membrane permeabilization, while damaging cell DNA without harming normal cells.	Breast cancer medication	-	[165]
≤10%	-	-	50–190 kDa	CS/Liquid paraffin/Tween 80/Span 80/magnesium stearate	Emulsification	-	Cinnamaldehyde	Round-shaped; d_TEM_ = 80–150 nm	Increased chemical stability and synergistic antibacterial action against Gram-positive and Gram-negative bacteria.	Medical textiles (e.g., wound dressings)	-	[89]
5%	-	-	-	CS/TPP	Ionic gelation	-	Vaccarin	Round-shaped; d_TEM_ ≈ 40 nm, d_DLS_ = 216.6 nm; ζ = 37.1 mV	No evidence of cytotoxic effects; increased umbilical vein endothelial cells proliferation and migration; up-regulated IL-1β and PDGF-BB factors, promoting angiogenesis.	Wound healing	Burst, then sustained release	[105]
≤15%	-	-	-	CS/TPP	Ionic gelation	*Pterocarpus marsupium* Roxburgh heartwood extract	-	Round-shaped; d_SEM_ = 400 nm; d_DLS_ = 676 nm	Inhibition against Gram-positive and Gram-negative bacteria. Healing of complicated surgical wounds.	Wound healing	Diffusion	[163]
15%	-	-	Low	CS/TPP	Ionic gelation	*-*	*Gallic acid*	Round-shaped; d_DLS_ = 117.5–356.6 nm; ζ = 18.3–33.6 mV	Accelerates angiogenesis, hexosamine synthesis, collagen deposition, and recruiting immune cells at wound area.	Wound healing dressings	pH-triggered desorption, then disintegration	[87]
-	-	-	-	CS/TPP/Tween 80	Ionic gelation and emulsification	-	*Pandanus tectorius* fruit extract	Round-shaped; d_DLS_ = 160.4 nm	No evidence of cytotoxic responses; increased SR-B1 gene expression required for an effective reduction of hypercholesterolemia-related symptoms.	Control medication for hypercholesterolemia	-	[150]
-	-	-	-	CS/TPP	Ionic gelation	-	Eugenol	Round-shaped; d_SEM_ = 23–16-37.67 nm; ζ = −49.6 mV	Reduced expression of TGF-β and MCP-1 genes; NPs revealed increased immunomodulatory, anti-inflammatory, and antioxidant potential.	Treatment of autoimmune diseases, such as rheumatoid arthritis	-	[166]
21%	-	-	206.4 kDa	CS/Tween 80	Emulsification and spray-drying	*-*	Lemongrass essential oil (LEO) and geranium essential oil (GEO)	Round-shaped; d_SEM_ = 4.959 and 5.009 µm; ζ = 45.26 and 47.34 mV, respectively	Higher thermal and colloidal stability than raw CS and EOs. The MIC for *C. albicans* was reduced up to 64 times. Reduced biomass of mature biofilm up to 84%.	Compounds that have antibiofilm activity against *C. albicans.*	Diffusion	[72]
91.2%	-	-	106.8 kDa	CS/TPP	Ionic gelation	Saussurea costus	-	Round-shaped; d_TEM_ = 48 nm; ζ = 3.28 mV	Notable antimycotic potentiality against all examined strains, with vigorous structural and morphological alterations.	Antimycotic agent to control resistant pathogenic yeast strains	-	[167]
9.6	-	-	100–300 kDa	CS/TPP	Ionic gelation	-	Cinnamon leaf oil	Round-shaped	Significant reduction of viable cells, right after 2 h of incubation.	*P. aeruginosa*’s infection control	pH-responsive release	[152]
17%	-	-	>150 kDa	CS/TPP	Ionic gelation	*Arrabidaea chica* extract	*-*	Round-shaped; d_TEM_ = 20–60 nm, d_DLS_ = 60–153 nm; ζ = 32.1–32.9 mV (Δ load content)	Gastroprotective effect. Biocompatibility, antiulcerogenic activity.	Ulcer-healing pharmaceutical systems	-	[168]
